# Construction and assessment of the immunogenicity and bactericidal activity of fusion protein porin A from *Neisseria meningitidis* serogroups A and B admixed with OMV adjuvant as a novel vaccine candidate

**DOI:** 10.22038/ijbms.2020.40470.9

**Published:** 2020-06

**Authors:** Parviz Afrough, Mohammad Reza Asadi Karam, Farzam Vaziri, Ava Behrouzi, Seyed Davar Siadat

**Affiliations:** 1 Department of Mycobacteriology and Pulmonary Research, Pasteur Institute of Iran, Tehran, Iran; 2 Microbiology Research Center (MRC), Pasteur Institute of Iran, Tehran, Iran; 3 Department of Molecular Biology, Pasteur Institute of Iran, Tehran, Iran; 4 Endocrinology and Metabolism Research Center, Endocrinology and Metabolism Clinical Sciences Institute, Tehran University of Medical Sciences, Tehran, Iran

**Keywords:** Adjuvant, Fusion protein, Neisseria meningitidis, Outer membrane vesicle, Porin A

## Abstract

**Objective(s):**

The porins A and B and also outer membrane vesicles (OMVs) of *Neisseria meningitidis* are used for vaccine purposes. In the present study, we aimed to design a new vaccine candidate based on a fusion of PorA of serogroups A and B of *N. meningitidis* admixed with OMV and evaluate it in an animal model.

**Materials and Methods:**

After bioinformatic studies, a fusion protein composed of porin A from both serogroups A and B of *N. meningitidis* was constructed, expressed, and purified by nickel resins. Extraction of OMV of *N. meningitidis* was performed using a chemical method. The mice were vaccinated subcutaneously in different groups with mixtures of PorA proteins, OMV, and Freund’s adjuvants. Then, the immune responses were measured using the ELISA method. Finally, serum bactericidal activity (SBA) procedure was applied to assay the activity of the immune responses in mice.

**Results:**

Mice received the PorA protein plus Freund’s adjuvant. Mice vaccinated with PorA fusion of serogroups A+B plus Freund’s adjuvant produced more IgG, IgG1, and IgG2a than combinations admixed with OMV. Furthermore, the vaccinated mice tended to direct the IgG responses toward IgG1. Sera of the mice that received PorA+Freund’s and those that received PorA+OMV produced higher bactericidal activity than the controls.

**Conclusion:**

Fusion protein porin A could be a valuable target for developing vaccines against *N. meningitidis*. Although, Freund’s adjuvant induced the strongest IgG responses, given that Freund’s adjuvant has no human use, and OMV is a human adjuvant, OMV could be considered in vaccine design against *N. meningitidis*.

## Introduction

*Neisseria meningitidis* is divided into 12 serogroups based on the structural differences of the polysaccharide capsule (1). A, B, C, Y, and W135 are major pathogenic serogroups in humans with varying geographic spread in different regions. For example serogroup A develops epidemics in Asia and Africa, serogroup C in Europe, and serogroup Y and W135 in the United States (2). A recent epidemic in New Zealand, related to serogroup B, showed the potential of this bacterium in the development of communicable diseases (3). Sporadic nature, abrupt onset, antibiotic resistance especially to ciprofloxacin, and rapid and severe progression of meningococcal disease suggest the need for vaccination as a useful way to manage the diseases caused by this human pathogen.

 The polysaccharide capsule is one of the major factors of meningococcal virulence that have been used to develop vaccines. There are currently two types of approved capsule-based vaccines: 1) bivalent or tetravalent polysaccharide vaccine for serogroups A, C, Y, and W135; 2) conjugate polysaccharide vaccine for serogroups A, C, Y, and W135. The serogroup B induces cross reaction due to the similarity of its polysaccharide capsule with human neuronal cell surface glycoproteins, and thus is a poor immunogen and forms autoantibodies. Accordingly, multiple studies have been carried out on the breakdown of immunological tolerance, the prevention of autoantibody formation and its replacement with induced bactericidal antibodies against serogroup B polysaccharide capsule, which did not yield satisfactory results (4, 5). Therefore, researchers have focused on the other components of the cell wall, in particular outer membrane vesicles (OMVs) (6-8). OMVs are small spherical forms taken from the cellular surface, secreted by bacteria to distort the immune system. Packaging of enzymes such as proteases and glycosidases as cargoes to OMVs plays a prominent role in the acquisition of nutrients for bacterial communities (9). These vesicles have compounds similar to outer membrane protein (OMP), and contain protein, lipid, and other membrane compounds (10). The outcomes of the OMV vaccines showed that OMP can induce protective antibodies against meningococcal serogroup B (11). Therefore, researchers have scanned the conserved outer membrane proteins by genomic analysis or reverse vaccinology and are trying to identify relatively conserved proteins using *in silico *studies to assess these proteins as potential vaccine candidates. The meningococcal outer membrane contains the homotrimeric proteins that form the pores of the bacterial cell wall and are expressed in all strains of bacterial pathogens (12). The porins are some of the most abundant proteins produced by meningococcal species and their differences with other OMPs are in their amino acid composition. Unlike other membrane proteins, the porins have polar properties and only beta strands in secondary structure of their proteins. Each subunit forming pores contains 250 to 450 amino acids, which form anti-parallel beta strands, so that all strands are linked to the adjacent strand with hydrogen bonds between the amino acids. These strands are bonded by a long loop structure at the outer membrane surface and a tight beta turn at the inner or periplasmic surface (12, 13). The porins A and B are among the most important meningococcal outer membrane proteins that have been used as vaccine candidates in various studies. Among them, the most bactericidal antibody response has been generated against porin A (PorA) (14). Furthermore, most of the OMV-based vaccines against meningococus have been currently designed on the basis of PorA, which has been selected in the present study. Thus, in the present study, we aimed to design a new vaccine candidate based on a fusion composed of PorA of *N. meningitidis *serogroups A and B admixed with OMV as adjuvant and assayed its immunogenicity and efficacy in a mice model.

## Materials and Methods

***Protein modeling***

 Nucleotide sequences of the *porA* gene in the standard strains of *N. meningitidis* serogroups A (ATCC13077) and B (ATCC13090) were obtained from NCBI database and the I-Tasser server, based on multidisciplinary modification for protein modeling, which showed 3D models and their confidence-score (C-score). According to the I-TASSER benchmark, greater C-score reflects better quality. The quality and reliability of the modeled structure were confirmed and evaluated using RAMPAGE and ProSa Web. Some of the properties of the primary structure, such as estimated half-life, aliphatic index, molecular weight, theoretical isoelectric point (PI), mean hydropathicity (GRAVY), instability index, and amino acid composition were obtained by the ProtAParam ExPASy tool.

***Preparation of fusion construct***

 The sequences of standard strains of serogroups A and B (ATCC13077 and ATCC13090, respectively), and pET28a vector were sent to BIOMATIC Co (Canada) for preparation of constructs. According to the protocol, the received lyophilized samples were solubilized in 100 μl of TE buffer. First, double enzyme digestion with *Nco*I and *Hind*III enzymes was performed and then the recombinant plasmid PorA (AB)-pET28a was transformed into *E. coli* BL21 (DE3) by the thermal shock method using CaCl_2_, and the recombinant clones were screened using Colony PCR with T7 specific primers.

***Induction, expression, and purification of PorA recombinant proteins***

To express the resulting recombinant proteins, the bacteria containing the recombinant plasmid were first inoculated into 5 ml of LB (Luria-Bertani, QUELAB) medium containing kanamycin (50 μg/ml) and incubated for 18 hr in a shaking incubator at 37 ^°^C. On the next day, 500 μl of these cultures were inoculated into 50 ml of LB broth medium and placed in the shaking incubator until absorbance (OD) of 0.5 at a wavelength of 600 nm. After that, obtained culture medium was allocated to 10-ml Falcon tubes and induced by IPTG (Isopropyl β-D-1- thiogalacto pyranoside) (Sigma-USA) at 0.5, 1, 2, and 4 mM for four hours, followed by OD measurement for determining the best concentration of IPTG with the highest level of protein expression. The cultures were centrifuged and the resulting sediment was used to check the expression of the proteins and then analyzed using SDS-PAGE and Coomassie blue staining. In addition, the Western Blot technique was applied to confirm the presence of protein.

***Western blot technique***

The purpose of the Western blot test (immunoblotting) was to confirm the expression and evaluation of antigenicity and immunological characteristics of recombinant PorA (rPorA). The proteins loaded on SDS-PAGE gel were transferred into Polyvinylidene Difluride (PVDF) membrane (Hi-bond Amersham Biosciences, USA) by using the wet-transfer system. Panceau S dye was used to control the transfer, and the membrane was sectioned into thin strips containing transferred proteins. Blocking of PVDF was performed with skim milk. In the next step, the PVDF membrane was incubated with Anti-6X His tag antibody conjugated to horseradish peroxidase (HRP) with a dilution of 1:6000 (Qiagen, USA). Washing was done with a Tris buffer salt containing 0.5% Tween 20 (TBS-T). The proteins appeared after adding 30% H_2_O_2_ and DAB (Sigma, USA).

***Purification of PorA recombinant protein***

 The pellet obtained from the previous steps was resuspended in a lysate buffer (8 M of urea, 0.1 M of NaH2PO4, and 0.01 M of Tris, pH=8.0). The resulting solution was sonicated 20 times with 25% power and each time for 20 sec with an interval of 20 sec and centrifuged at 10,000 rpm for 15 min. The resulting sediment was dissolved in a binding buffer, shacked for one hour, and transferred to a column containing Ni-nitrilotriacetic acid (Ni-NTA) agarose. After one hour of shaking, the column was washed several times with washing buffer and finally the bounded proteins were eluted with addition of elution buffer. Then, the quality of the purified proteins was evaluated by SDS-PAGE. The Bradford protein assay also was used to calculate the concentration of the purified recombinant proteins.

***Measurement of recombinant endotoxin level using Limulus Amebocyte Lysate (LAL) assay***

The use of chromogenic substrate and HRP lysate is the basis of LAL test (according to the Thermo Kit protocol). In this experiment, 0.1 ml of the sample was mixed with 0.1 ml of LAL, incubated for 8 min at 37 ^°^C, added 0.1 ml of glacial acetic acid to stop the reaction, and read using spectroscopy to obtain OD at 405 nm. Finally, the endotoxin value was calculated using a standard curve.

***Extraction and purification of OMV***

The OMV from *N. meningitidis* serogroup B strain CSBPI G-245 was used as adjuvant; this method has already been described (15, 16). In summary, in this study, OMV was obtained from stress in bacteria caused by detergents such as EDTA and sodium deoxycholate and using different revolutions of ultracentrifugation. The OMV deposit was dissolved in 15 ml of sucrose 3% and sterilized by passing it through 0.25-micron filter. The protein content was measured by NanoDrop. Moreover, 12% gel and standard markers (Sigma, USA) were used to estimate the molecular weight of proteins presented in OMVs. The chromogenic LAL method based on Thermo Kit was used to measure the endotoxin level in the OMV samples. Ultimately, the morphological characteristics of OMV were confirmed by negative contrast staining with potassium phosphotungstate and examined under a TEM microscope (Carl Zeiss, Germany).

***Mice immunization schedules and blood collection***

For animal testing, female BALB/c mice 6 to 7 weeks of age were procured from the Pasteur Institute of Karaj, Iran and divided into 9 groups of 5. Subcutaneous injection was performed in three doses with two-week intervals (days 0, 14, and 28). Additionally, the OMV from serogroup B strain G245 and Freund’s complete and incomplete were used as adjuvants. The final volume of injection was 100 μl and contained 25 μg of each protein. Furthermore, OMV was used at the final concentration of 1.08 mg/ml in each preparation. The blood samples were taken from mice at 0, 14, 28, and 42 day intervals after first vaccine dose. Sera were collected and stored in a refrigerator at 4 ^°^C.

***ELISA assay***

 Initially, 100 μl of PorA prepared in PBS 1x was added to 96-well plates (at a concentration of 1 μg per well in 100 μl of PBS 1x). The plates were incubated at 4 °C overnight to coat the antigen on the plate bottom. By adding 300 μl of bovine serum albumin (BSA 3% in PBS 1x), the plates were blocked for an hour at room temperature, washed three times with washing buffer (PBS 1x + Tween 20), dried, added 100 μl of mice sera at a dilution of 1:1000 and incubated for two hours at room temperature. After washing and drying the plates, 100 μl of anti-IgG conjugate antibody (Sigma, USA) were added at a dilution of 1:6000. After incubation for two hours at room temperature, the plates were washed 5 times and 100 μl of TMB chromogenic substrate (Razi Tab, Iran) was added. About 30 min after the appearance of color, 100 μl of 2N sulfuric acid was added as a stopper to the plates. The plates were read in an ELISA microplate reader at a wavelength of 450 nm. IgG1 and IgG2a isotypes were also evaluated according to the protocols.

***Serum Bactericidal Activity (SBA) procedure***

The procedure of serum bactericidal activity (SBA) has already been described (17). In brief, safe and decomplemented mice sera were distributed using sterile normal saline diluted with serial dilutions inside polystyrene microplates. Then, 25 μl of serum, 12.5 μl of bacterial suspension containing 10^5^ CFU/ml, and 12.5 μl of baby rabbit serum (as an external complement source) were added into each well. The colonies were counted at time zero (t_0_) and one hour after incubation (t_1_). The titer in each series was equal to serum dilution in which the count of colonies grown at t_1_ showed a decrease equal to or greater than 50%.

***Statistical analysis***

Differences between induced immune response and immune response functions in each mouse group were analyzed using one-way ANOVA, Student’s t-test, two-way ANOVA, and repeated measurements, and *P-value* <0.05 was considered as a statistically significant difference between the groups. 

## Results

***Protein modeling***

**Figure 1:**
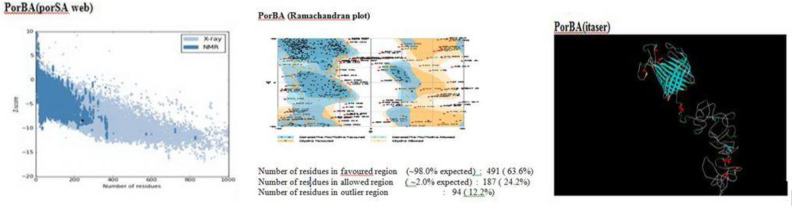
Protein modeling. Modeling of fusion protein was done by I-Tasser server, prosa, and ramachandran plot. Z score in Prosa was −8.52. Ramachandran plot of fusion protein showed the number of residues in favored, allowed, and outer regions were 63.6%, 24.2%, and 12.2%, respectively

The 3D structure and protein modeling were detected by the I-Tasser server. The results showed that the C-scores were +1.28, +0.96, and +0.56 for PorA fusion protein, serogroup A, and serogroup B, respectively. A Ramachandran plot also showed 98% PorA residues within appropriate area. Validation of the 3D structure with Prosa web indicated that the Z-score value of the fusion protein (-8.52) was closer to the range of native conformation analyzed by crystallization (Figure 1).

***Induction and expression of PorA recombinant proteins***

**Figure 2:**
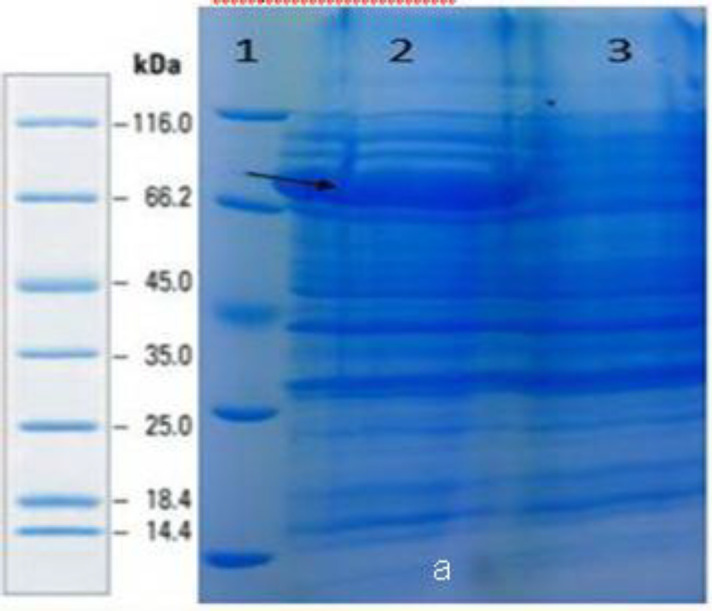
SDS-PAGE analysis of the fusion protein PorA. 1) marker, 2)fusion protein, 3) negative control

 After inducing the expression of proteins in the pET28a vector at different times, the expression of the proteins was assessed using SDS-PAGE (Figure 2). The PorA proteins showed the highest expression at 1 and 2 mM IPTG concentrations for serogroups A and B, respectively and IPTG 2 mM for expression of fusion protein at 4 hr incubation time. The final confirmation of protein expression was performed by Western blot.

***Confirmation of the purified proteins using Western blot***

**Figure 3:**
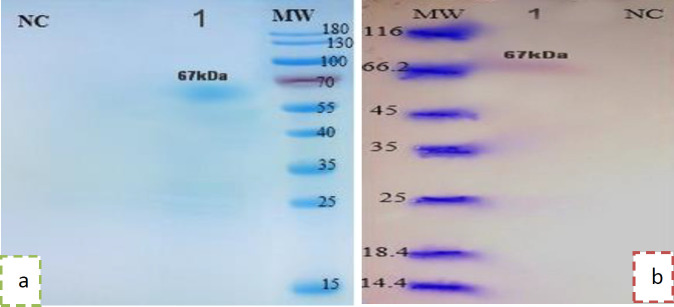
a: Purified expressed fusion protein 1) fusion protein, NC) negative control b: Western blot analysis of the fusion protein PorA. MW) marker, 1) fusion protein, NC) negative control

 After purification of recombinant proteins using Ni-NTA resins, the results of SDS-PAGE and Western blot showed that the purified proteins associated with serogroups A and B were presented in the band of 45 kDa and a 67 kDa band for fusion protein that indicated the accuracy of purified expressed proteins (Figure 3). The purified recombinant endotoxin levels were 3.8 EU/ml, 6.2 EU/ml for serogroup A and serogroup B, respectively, and 5.3 EU/ml for fusion protein and 0.08 EU/ml for OMV. The Bradford results indicated that the concentration of PorA protein was about 250 and 300 μg for the serogroups A and B, 1.02 mg for OMV, and 400 μg for the fusion protein.

***Extraction of OMV from N. meningitidis serogroup B and microscopic findings***

**Figure 4:**
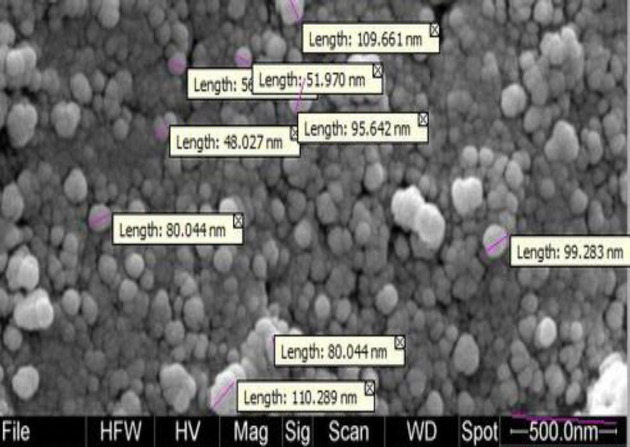
OMV electron micrograph of the *Neisseria meningitidis *serogroup B strain CSBPIG245

 The level of LPS in OMV was within the acceptable range for injection (0.8 EU/ml). The obtained OMV protein content was 1.02 mg/ml using the Bradford protein assay. Electrophoretic mobility pattern of OMV on SDS-PAGE (12% gel) and electron microscopic micrographs confirmed the presence of OMV (Figure 4).

***Evaluation of the specific antibody response of IgG and IgG isotypes***

**Figure 5:**
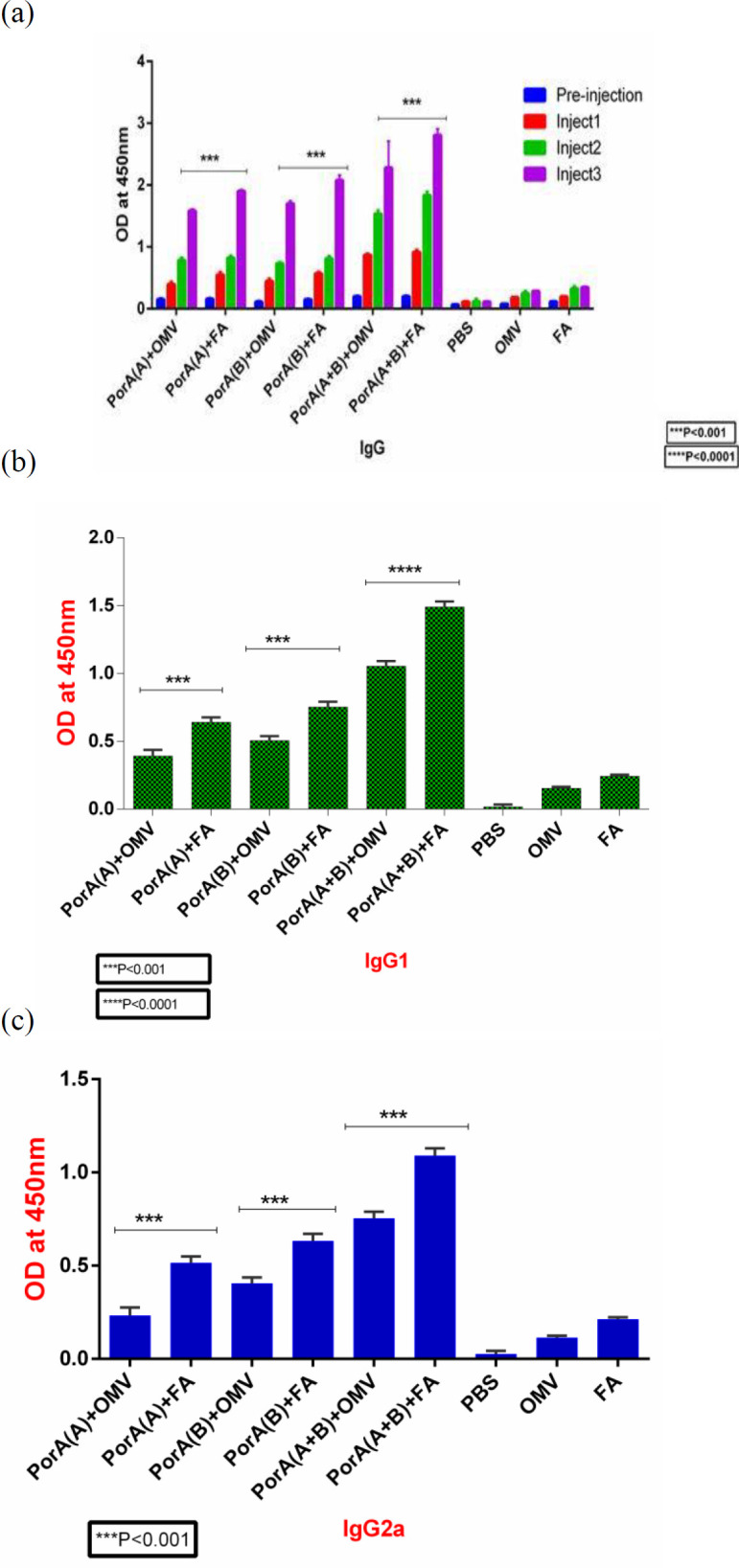
Analysis of IgG and IgG isotypes in the vaccinated mice. The mice groups were immunized with different vaccine combinations of PorA, freund, and OMV adjuvants. After three immunizations, (a) total IgG, (b) IgG1, and (c) IgG2a levels were measured in the mice by ELISA test at serum dilution of 1:1000. The IgG responses in mice groups receiving all PorA proteins plus freund's adjuvant were significantly higher compared with mice receiving PorA admixed with OMV. The results are shown as the mean optical density±SD from 5 mice per groups at a wavelength of 450 nm

OMV: outer membrane vesicles; FA:Freund’s adjuvant; PBS: Phosphate Buffered Saline; PorA: porin serogroup A; PorB: porin serogroup B

 The sera of immunized mice were diluted 1:1000 and the serum concentration of antibodies was investigated with indirect ELISA. We used repeated measurements to compare the mean OD of sera in the immunized groups on days 14, 28, and 42, one-way ANOVA test was used to test pairwise comparison of the groups on day 42 as well. It was observed that after first vaccination, all vaccine combinations with Freund’s or OMV adjuvants produced higher IgG, IgG1, and IgG2a responses compared with control mice receiving PBS, OMV, or Freund’s adjuvant alone (*P*<0.001). Furthermore, the first and second booster vaccines could significantly improve IgG and IgG isotype responses in all mice compared with the first vaccine dose (*P*<0.01). The total IgG and IgG isotypes in the groups receiving PorA protein (serogroups A or B) plus Freund’s adjuvant were enhanced compared with those receiving PorA + OMV and this difference was statistically significant (*P*<0.0001). The mean OD difference was also significantly increased in the groups receiving PorA fusion protein of serogroups A and B plus Freund’s adjuvant in comparison with the groups receiving PorA fusion protein of serogroups A and B plus OMV (*P*<0.0001) (Figures 5a, 5b, and 5c). Overall, it was observed that the levels of IgG1 responses in all groups immunized with PorA (A or B) with or without adjuvants were higher than IgG2 levels. Our results also showed that mice immunized with PorA (serogroup B) in the presence or absence of adjuvant induced more IgG1 and IgG2a than mice receiving PorA (serogroup A) (Figures 5b and 5c). 

***The SBA measurement***

**Figure 6:**
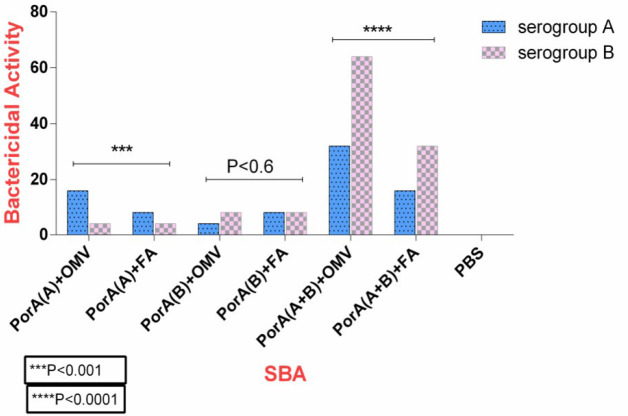
The bactericidal antibody titer against *Neisseria* *meningitidis *serogroups A and B in different mice groups. Mice were immunized three times with different formulations and the SBA measurement was performed in the sera of mice after the third vaccination. The bactericidal antibody titer in groups vaccinated with antigens admixed with OMV was significantly higher compared with mice receiving antigens plus Freund’s adjuvant

OMV: outer membrane vesicles; FA:Freund’s adjuvant; PBS: Phosphate Buffered Saline; PorA: porin serogroup A; PorB: porin serogroup B

Anti-sera were evaluated *in vitro* for bactericidal activity via complement against *N. meningitidis* strains. The sera of the mice belonging to the group receiving PorA protein plus Freund’s adjuvant, and those receiving PorA protein plus OMV adjuvant showed greater bactericidal activity compared with the control group. The highest activity in these two groups was observed in the serum dilutions of 1:32 and 1:64. The two groups in these dilutions showed bactericidal activity higher than 50% compared with the control group (Figure 6). The results of this study showed that the difference in bactericidal antibody titer on day 42 between the groups receiving PorA serogroups A and B with OMV or Freund’s adjuvants was significant compared with the controls (*P*<0.0001) . The bactericidal antibody levels in mice groups receiving each of these antigens plus OMV was significantly higher in comparison with the groups receiving antigen plus Freund’s adjuvant (*P*<0.001). The highest bactericidal activity against serogroup B was observed in the sera of mice receiving fusion protein of PorA (A + B) + OMV protein (up to the serum dilution of 1:64 ), and the most bactericidal activity against serogroup A was related to the sera of mice receiving fusion protein of PorA (A + B ) + OMV (up to the serum dilution of 1:32); this difference was statistically significant compared with the groups receiving fusion protein plus Frund’s adjuvant (*P*<0.0001).

OMV: outer membrane vesicles

## Discussion

Porin Class I or PorA with a molecular weight of 42-45 kDa is an intramembrane cationic protein, which is expressed in all strains and found in the main composition of the meningococcal outer membrane (18). In a topological study, this protein consisted of 16 parallel amphipathic beta chains (which cross the cell membrane), relatively conserved amino acid sequences between the strains, and eight extracellular hydrophilic loops. Two loops have potent immunogenic activity (loop1 and loop 4) that stimulate immune responses and induce antibody production. In fact, the epitopes in the loops 1 and 4, which contain variable domains 1 and 2 (VR1 and VR2), cause the production of bactericidal and opsonizing antibodies. The difference in PorA protein between strains, which is the principle for the subserotyping of strains, is related to loops 1 and 4 with variable domains of VR1 and VR2 (14, 19). In various studies, bactericidal anti-PorA antibodies have been shown to have more protective effects than other antigens. Therefore, most recent studies on vaccine candidates against *N. meningitidis* were based on the recombinant PorA (20, 21). Nevertheless, it has been shown that anti-PorA antibodies alone have limitations related to the induction of specific immunity against the corresponding subserotype. One way to overcome this problem is to use multivariate vaccines, so that hexavalent vaccines (containing 6 PorA subtypes) evaluated in the Netherlands cover 80% of European strains (22). Moreover, clinical trials on these vaccines have confirmed their efficacy and safety. In addition to producing bactericidal antibody, OMV-based vaccines containing *N. meningitidis* serogroup B PorA can create long-term immunological memory after injecting three doses of the vaccine (23). One of the benefits of OMV-based vaccines is the simpler and cheaper production of these types of vaccines compared with conjugate vaccines (24, 25). 

Pizza *et al. * analyzed the sequences of DNA fragments using computerized algorithms or the reverse vaccinology method to identify ORFs encoding outer membrane proteins, lipoproteins, secretory proteins, and proteins involved in pathogenesis. In this study, it was found that 29 proteins are able to induce antibacterial antibodies. One of these antigens is PorA, the antibody produced against which has shown very high bactericidal activity. The results of this study showed that recombinant PorA protein plus Freund’s adjuvants and OMV promote antibody responses and stimulate both bactericidal and opsonic activities (26).

 To date, one of the problems in the design of vaccines, especially vaccines based on recombinant proteins, is poor immunogenicity, for improvement of which adjuvants should be used (27). One of the other existing technologies is the production of fusion protein-based vaccines. This approach, unlike monovalent technology, has proven to be more likely to produce extensive protective immunity against meningococcal strains. The use of fusion protein technology allows the use of two different antigens that play important roles in the development of meningococcal infections (28).

The results of this study revealed that serum IgG antibodies against PorA protein of *N. meningitidis* serogroups A and B increased significantly in the immunized groups compared with the control groups. The serum IgG antibody levels significantly increased in the groups receiving PorA plus Freund’s adjuvant compared with the groups receiving PorA plus OMV. The results also revealed that the serum IgG antibody levels in the groups receiving fusion protein PorA (A + B) + FA increased significantly more compared with those in the other groups as well as in the group receiving fusion protein PorA (A + B) + OMV. Indeed, Freund’s adjuvant, as one the strongest adjuvants, stimulated more IgG responses compared with OMV, but given that Freund’s adjuvant has no human use, and because OMV is part of the bacterial structure, properly stimulates bactericidal antibody responses and opsonic activity, and has human use, it could be a superior adjuvant compared with Freund’s. In line with our study, various studies have shown that the vaccines containing polyvalent antigens have more protective effects than monovalent antigens, because the studied hybrid proteins plus the adjuvant with greater antibody response were able to induce more opsonic and bactericidal activities (29, 30).

Filatova *et al.* (2008) examined the protective properties of peptide fragments of conserved outer membrane proteins of OpaB, NspA, and PorA serogroup B in a mouse model. They reported that these peptides had a protective effect in the infected mice with homologous and heterologous strains of serogroup B. Subcutaneous injection of these compounds plus Freund’s adjuvant resulted in increased levels of serum IgG antibodies in vaccinated mice. These synthetic peptides showed a protective effect in the mice immunized with homologous and heterologous *N. meningitidis* strains of serogroups A and B and had more protective effect when synthetic peptide mixtures were used. In the present study, the hybrid proteins used in both serogroups stimulated more antibody levels than the proteins alone (31).

 In the present study, the levels of IgG1 and IgG2a isotypes and their ratio in the immunized mice were measured to evaluate the humoral and cell-mediated immune responses. The results showed that the serum IgG1 and IgG2a antibody levels against PorA proteins in the immunized groups increased significantly compared with the control groups. Meanwhile, the levels of these antibodies were higher in the groups receiving hybrid protein plus Freund’s adjuvant, and the highest response rate of these antibodies was related to the group receiving PorA hybrid protein (A + B) + FA. Meanwhile, the IgG1/IgG2a ratio in the studied proteins was more than 1.3. Therefore, given the increased IgG1 level, it can be indicative of the predominance of humoral responses (Th2) over cell-mediated responses (Th1). The immunity against *N. meningitidis* depends on the induction of bactericidal antibody responses. Hence, the SBA measurement is the most important and valuable test in the immunological evaluation of *N. meningitidis *(32, 33). The results of this study showed that the antibacterial activity produced against PorA proteins increased significantly in the immunized groups compared with the control groups. This increase was higher in the groups receiving antigens plus OMV adjuvant compared with those receiving antigens plus Freund’s adjuvant, while the bactericidal activity was higher in the groups receiving PorA (A + B) + OMV compared with all other groups. In other words, the SBA and OPA activities were higher in the groups receiving hybrid protein compared with those receiving proteins alone. In addition, the results revealed that these activities were higher against serogroup B compared with serogroup A. Finally, the serum bactericidal antibody titer in groups receiving PorA (A + B) + OMV was detected up to the dilution of 1:64 in the serogroup B and up to the dilution of 1:32 in the serogroup A. 

## Conclusion

Considering that OMV is a segment of the bacterial structure and stimulates specific bacterial antibody responses (reasonable and significant results) in addition to having human use approval, it can be used as an appropriate adjuvant. This study indicated that the polyvalent (hybrid) vaccines have better and more protective effects than monovalent ones. Moreover, the recombinant PorA protein plus OMV adjuvant were able to induce antibacterial antibody responses against serogroups A and B in the BALB/c mouse model.
